# Supplementary material for: High Serum Levels of Toxin A Correlate with Disease Severity in Patients with Clostridioides difficile Infection
Source: Antibiotics (Basel). 2021 Sep 10;10(9):1093. doi: 10.3390/antibiotics10091093 (PMC8470527; doi:10.3390/antibiotics10091093)
Supplement: Supplementary file 1 [file antibiotics-10-01093-s001.zip › antibiotics-1325685-supplementary.pdf]

Supplementary Materials

High Serum Levels of Toxin A Correlate with Disease Severity in Patients with *Clostridioides difficile* Infection

Guido Granata [1,†], Davide Mariotti [1,2,†], Paolo Ascenzi [2,3], Nicola Petrosillo [1,‡] and Alessandra di Masi [2,*,‡]

1 Clinical and Research Department for Infectious Diseases, National Institute for Infectious Diseases L. Spallanzani, IRCCS, 00149, Italy; guido.granata@inmi.it (G.G.); davide.mariotti16@gmail.com (D.M.); nicola.petrosillo@inmi.it (N.P.)
2 Department of Science, Section of Biomedical Sciences and Technologies, Roma Tre University, 00146 Rome, Italy; paolo.ascenzi@uniroma3.it
3 Accademia Nazionale dei Lincei, 00165, Rome, Italy
* Correspondence: alessandra.dimasi@uniroma3.it; Tel.: +39-06-57336363
† These authors contributes equally to this work.
‡ Co-last Authors.

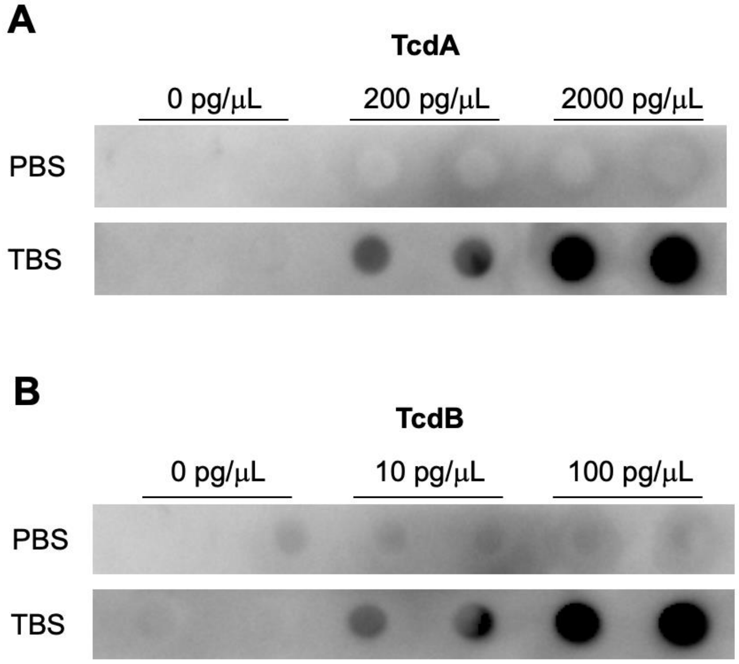

Figure S1. ***C. difficile*** **toxins resuspension in either PBS or TBS is critical for dot-blot sensitivity.** Representative images of dot-blot experiment using the commercial (**A**) TcdA and (**B**) TcdB toxins resuspended in either PBS or TBS. The resuspension in TBS allowed the solubilization of the serum lipid and consequently a higher sensitivity of the technique. Each toxin concentration was spotted on PVDF membrane in duplicate. Signals were revealed using specific anti-TcdA or anti-TcdB antibodies.

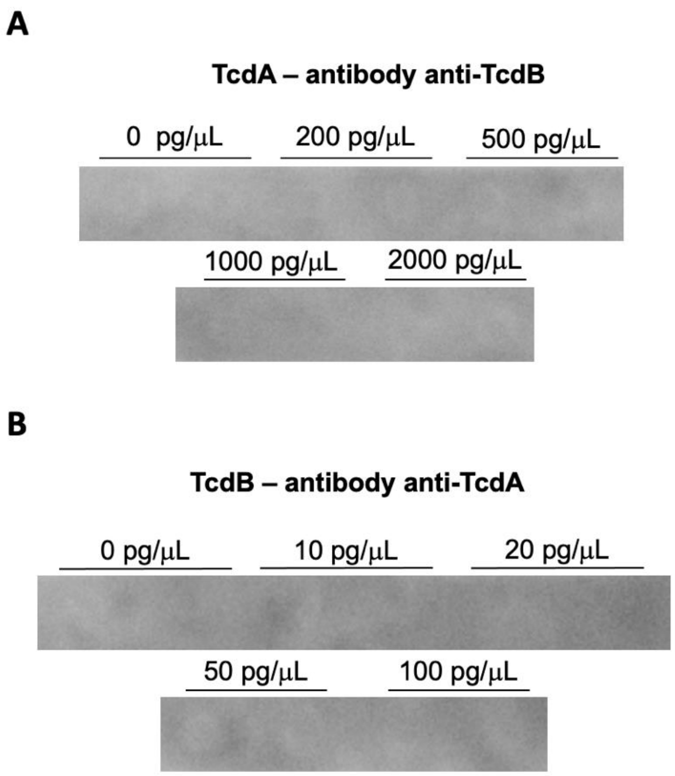

Figure S2. Cross-reactivity experiments to evaluate the specificity of the dot-blot assay. (**A**) The indicated concentrations of the commercial TcdA were spotted in duplicate and the PVDF filter was blotted using the anti-TcdB specific antibody. (**B**) The indicated concentrations of the commercial TcdB were spotted in duplicate and the PVDF filter was blotted using the anti-TcdA specific antibody.

Table S1. Demographic and epidemiological data, comorbidities, clinical characteristics, and outcome of the 35 CDI cases included in the study. CCI: Charlson Comorbidity Index; rCDI: recurrent CDI; COPD: chronic obstructive pulmonary disease. SD: standard deviation.

**Characteristics**	**Mean ± SD (%)**
Female gender	18 (51.4%)
Age (years)	60 (range: 19-86)
First CDI episode	30 (85.7%)
Recurrence of CDI	5 (14.3%)
No comorbidities	10 (28.6%)
Comorbidities	25 (71.4%)
Cardiovascular disease	13 (37.1%)
Heart failure	4 (11.4%)
Diabetes	7 (20.0%)
Renal failure	3 (8.6%)
Dialisis	0
Chronic liver failure	2 (5.7%)
Neurological disease	4 (11.4%)
Vasculitis	2 (5.7%)
COPD	9 (25.7%)
Solid cancer	3 (8.6%)
Blood cancer	3 (8.6%)
Transplant, immunodeficiency, immunosuppression	7 (20.0%)
Chronic inflammatory bowel disease	2 (5.7%)
Other concomitant infections	13 (37.1%)
Mean age-adjusted CCI at admission	3.6 (range: 0-8)
Antibiotic in the previous two months	32 (91.4%)
Antiacids in the previous two months	27 (77.1%)
Statins in the previous two months	4 (11.4%)
Steroids in the previous two months	13 (37.1%)
CDI severity	
Mild-moderate	17 (48.6%)
Severe	18 (51.4%)
Patients outcome	
Deceased before the discharge	1 (2.9%)
Recovered at home, no subsequent rCDI	28 (82.4%)
Subsequent rCDI	5 (14.7%)
Deceased, rCDI-related	0
Deceased, no rCDI-related	1 (2.9%)

Table S2. Definitions of CDI, microbiological evidence of CDI, CDI recurrence, mild CDI and severe CDI adopted in the study.

**CDI:** A clinical picture characterized by diarrhea or ileum or toxic megacolon in the presence of microbiological evidence of CDI.
**Microbiological evidence of CDI:**<br>- GDH positive antigen + A/B positive toxins (enzyme immunoassay).<br>- GDH positive antigen, A/B negative toxins (immunoenzymatic method) + positivity of the amplification of the genes that encode C. difficile toxins.<br>- Positivity of the amplification of the genes that encode C. difficile toxins + A/B positive toxins by enzyme immunoassay.<br>**Recurrence of CDI**: The onset of a new episode of CDI after at least two days from the resolution of the diarrhea and after the end of the antimicrobial treatment of the previous CDI episode.<br>**Mild CDI**: CDI in the absence of the following criteria: fever (> 38.5°), chills, hemodynamic instability, signs of ileus or peritonitis, leukocytosis (leukocytes >15,000 cells/μl) creatininemia increase >1.5 times the values before the infection, increase in serum lactates, histological evidence of pseudo-membranous colitis, radiological evidence of ileus or ascites.<br>**Severe CDI**: CDI in the presence of at least one of the following criteria: fever (> 38.5°), chills, hemodynamic instability, signs of ileus or peritonitis, leukocytosis (leukocytes >15,000 cells/μl) creatininemia increase >1.5 times the values before the infection, increase in serum lactates, histological evidence of pseudo-membranous colitis, radiological evidence of ileus or ascites.

Table S3. TcdA and TcdB plasma levels and presence of the binary toxin *tcdC* gene in the CDI strains found in the 35 patients enrolled in the study. PCR: polymerase chain reaction.

	**T0**		**T4**		**T10**		**Presence of the binary toxin *tcdC* gene by PCR**	**Infection Sever-ity**
# Patient	**TcdA (pg/μL)**	**TcdB (pg/μL)**	**TcdA (pg/μL)**	**TcdB (pg/μL)**	**TcdA (pg/μL)**	**TcdB (pg/μL)**		
001	79,8 ± 5,8	0,0	24,6 ± 5	0,0	14 ± 0,8	0,0	n/a	Mild
002	0,0	51,2 ± 0,6	0,0	49,6 ± 1,2	0,0	45,2 ± 6	n/a	Mild
003	112,4 ± 14,6	44 ± 1,6	0,0	22 ± 0,2	7,4 ± 1,6	19 ± 2,2	Negative	Mild
004	214,8 ± 4	137,4 ± 1	139,6 ± 3,2	120 ± 0,8	0,0	77,4 ± 4,8	n/a	Severe
005	126 ± 10	0,0	234 ± 3,8	0,0	231,4 ± 68,6	0,0	n/a	Severe
006	258,8 ± 4,8	0,0	265 ± 36	0,0	227,8 ± 1,2	0,0	n/a	Severe
007	243 ± 7	0,0	239 ± 1	0,0	217 ± 30	0,0	Positive	Mild
008	256,8 ± 53,6	0,0	175,2 ± 4,8	0,0	192,4 ± 3	0,0	n/a	Mild
009	300 ± 82	0,0	178,6 ± 12,4	0,0	n/a	n/a	n/a	Mild
010	257 ± 34	0,0	69 ± 10,4	0,0	17,4 ± 0,6	0,0	Negative	Mild
011	220 ± 30	0,0	104 ± 10	0,0	32,6 ± 6	0,0	n/a	Mild
012	18,6 ± 26	0,0	52,8 ± 38	0,0	54 ± 18	0,0	Negative	Severe
013	38,4 ± 20	0,0	30,4 ± 22	0,0	16,4 ± 10	0,0	n/a	Mild
014	28,6 ± 16	0,0	0,0	0,0	0,0	0,0	Negative	Severe
015	25,4 ± 0,8	0,0	17,6 ± 12	0,0	0,0	0,0	Positive	Severe
016	70,8 ± 26	0,0	58,4 ± 42	0,0	15,6 ± 0,06	0,0	n/a	Mild
017	117,8 ± 54	0,0	85,8 ± 29	0,0	38,8 ± 30	0,0	n/a	Severe
018	70,8 ± 2,2	0,0	11,6 ± 8	0,0	0,0	0,0	Negative	Severe

019	137,2 ± 22	0,0	192,2 ± 4,8	0,0	23,2 ± 16	0,0	n/a	Severe
020	28,8 ± 7	0,0	98,6 ± 24,6	0,0	22 ± 14	0,0	Positive	Severe
021	0,0	0,0	34 ± 2,6	0,0	21,6 ± 7,4	0,0	Positive	Mild
022	0,0	0,0	0,0	0,0	0,0	0,0	n/a	Mild
023	20 ± 14	0,0	57,8 ± 19,2	0,0	28,6 ± 20	0,0	n/a	Severe
024	0,0	0,0	0,0	0,0	0,0	0,0	Positive	Mild
025	n/a	n/a	25,8 ± 0,4	0,0	0,0	0,0	n/a	Mild
026	0,0	4 ± 0,4	55,6 ± 16	12 ± 7,2	84,6 ± 15	90,6 ± 3,8	n/a	Severe
027	0,0	0,0	65,6 ± 30	0,0	58,4 ± 5,2	0,0	n/a	Severe
028	26,8 ± 10,8	0,0	78,4 ± 26	0,0	78 ± 17	0,0	Positive	Severe
029	0,0	0,0	30 ± 2,6	0,0	n/a	n/a	Positive	Mild
030	113,4 ± 5,4	0,0	122,4 ± 11,4	0,0	17,8 ± 12	0,0	Negative	Mild
031	40,2 ± 15,6	0,0	109 ± 12,4	0,0	66,8 ± 12,2	0,0	n/a	Mild
032	10,8 ± 4,4	0,0	38,8 ± 15,2	0,0	33 ± 0,6	0,0	Negative	Severe
033	0,0	0,0	n/a	n/a	n/a	n/a	Positive	Mild
034	196,6 ± 10	0,0	150 ± 22	0,0	n/a	n/a	n/a	Severe
035	124,6 ± 13	0,0	103,8 ± 23,8	0,0	n/a	n/a	n/a	Mild

Table S4. Risk factors for high level of TcdA toxemia (TcdA > 60 pg/ L) at the CDI onset. RR: risk ratio. CI: confidence interval. SD: standard deviation. CCI: Charlson Co-morbidity Index.

	**TcdA ≤ 60 pg/ L at T0 (N = 17)**	**TcdA > 60 pg/ L at T0 (N = 17)**	**RR (95% CI)**	**Fisher's test***
Female gender	8 (47.0%)	9 (52.9%)	1.1 (0.5-2.2)	P = 1
Mean age (years)	58.5	59.3	-	P = 0.7
Mean age-adjusted CCI at admission ±SD	3.4 ± 2.5	3.7 ± 3.3	-	P = 0.9
Comorbidities				
No comorbidities	5 (29.4%)	5 (29.4%)	1.0 (0.4-2.1)	P = 1
Cardiovascular disease	5 (29.4%)	8 (47.0%)	1.4 (0.6-3.2)	P = 0.4
Heart failure	0 (0%)	4 (23.5%)	-	P = 0.1
Diabetes	3 (17.6%)	4 (23.5%)	1.2 (0.4-3.0)	P = 1
Renal failure	3 (17.6%)	0 (0%)	-	P = 0.2
Inflammatory bowel disease	2 (11.7%)	0 (0%)	-	P = 0.4
Chronic liver failure	2 (11.7%)	0 (0%)	-	P = 0.4
Neurological disease	2 (11.7%)	2 (11.7%)	1 (0.3-2.8)	P = 1
Vasculitis	1 (5.8%)	1 (5.8%)	1 (0.2-4.1)	P = 1
COPD	6 (35.2 %)	2 (11.7%)	0.5 (0.3-1.0)	P = 0.2
Solid cancer	2 (11.7%)	1 (5.8%)	0.7 (0.3-1.7)	P = 1
Blood cancer	1 (5.8%)	2 (11.7%)	1.5 (0.3-7.9)	P = 1
Transplant, immunodeficiency, immunosuppression	3 (17.6%)	4 (23.5%)	1.2 (0.4-3.0)	P = 1
Other concomitant infections	6 (35.2 %)	7 (41.1%)	1.1 (0.5-2.3)	P = 1
Laboratory findings before CDI diagnosis				
Basal Albumin (g/dL ±SD)	3.3 ± 0.5	3.9 ± 0.6	-	P = 0.02
Basal Creatinine (mg/dL ±SD)	0.7 ± 0.2	0.7 ± 0.2	-	P = 0.4
Laboratory findings at T0				
White blood cell peripheral count (10^3cells/µL ±SD)	11.38 ± 5.71	11.38 ± 6.47	-	P = 0.8
Neutrophils peripheral count (10^3cells/µL ±SD)	8.02 ± 5.33	8.66 ± 5.71	-	P = 0.7
Creatinine (mg/dL ±SD)	1.0 ± 0.7		-	P = 0.7

Albumin (g/dL ±SD)	3.2 ± 0.5	3.5 ± 0.6	-	P = 0.1
TcdB (ng/mL ±SD)	0.15 ± 0.60	0.52 ± 1.7	-	P = 0.4
Severe CDI	6 (35.2 %)	12 (70.5%)	2.0 (1-4.2)	P= 0.04
Patients outcome				
Deceased	0 (0%)	1 (5.8%)	-	P = 1
rCDI	3 (17.6%)	2 (11.7%)	0.8 (0.3-1.8)	P = 1

Table S5. Risk factors for detectable level of TcdB toxemia (TcdB > 4 pg/μL) at the CDI onset. RR: risk ratio. CI: confidence interval. SD: standard deviation. CCI: Charlson Co-morbidity index.

	**TcdB not detectable at T0** ***(N = 30)***	**TcdB > 4 pg/mL at T0** ***(N = 4)***	***RR (95% CI)***	***Fisher's test****
Female gender	15 (50.0%)	2 (50.0%)	1.0 (0.7-1.2)	P = 1
Mean age (years)	59.8	52.7	-	P = 0.5
Mean age-adjusted CCI at admission ±SD	3.6 ± 2.9	3.0 ± 3.5	-	P = 0.6
Comorbidities				
No comorbidities	8 (26.6%)	2 (50.0%)	2.4 (0.3-14.7)	P = 0.5
Cardiovascular disease	11 (36.6%)	2 (50.0%)	1.0 (0.8-1.4)	P = 0.6
Heart failure	3 (10.0%)	1 (25.0%)	1.2 (0.6-2.1)	P = 0.4
Diabetes	6 (20.0%)	1 (25.0%)	1.0 (0.7-1.4)	P = 1
Renal failure	3 (10.0%)	0 (0%)	-	P = 1
Inflammatory bowel disease	2 (6.6%)	0 (0%)	-	P = 1
Chronic liver failure	2 (6.6%)	0 (0%)	-	P = 1
Neurological disease	4 (13.3%)	0 (0%)	-	P = 1
Vasculitis	2 (6.6%)	0 (0%)	-	P = 1
COPD	8 (26.6%)	0 (0%)	-	P = 0.5
Solid cancer	3 (10.0%)	0 (0%)	-	P = 1
Blood cancer	3 (10.0%)	0 (0%)	-	P = 1
Transplant, immunodeficiency, immunosuppression	7 (23.3%)	0 (0%)	-	P = 0.5
Other concomitant infections	12 (40.0 %)	1 (25.0%)	0.9 (0.7-1.1)	P = 1
	Laboratory findings before CDI diagnosis			
Basal Albumin (g/dL ±SD)	3.6 ± 0.6	3.2 ± 0.3	-	P = 0.3
Basal Creatinine (mg/dL ±SD)	0.7 ± 0.2	0.6 ± 0.1	-	P = 0.2
	Laboratory findings at T0			
White blood cell peripheral count (10^3cells/μL ±SD)	11.00 ± 5.85	12.65 ± 7.94	-	P = 0.6
Neutrophils peripheral count (10^3cells/μL ±SD)	8.11 ± 5.29	10.12 ± 7.17	-	P = 0.4
Creatinine (mg/dL ±SD)	1.0 ± 0.6	0.8 ± 0.4	-	P = 0.4
Albumin (g/dL ±SD)	3.4 ± 0.5	3.2 ± 0.6	-	P = 0.5
TcdA (ng/mL ±SD)	4.68 ± 4.84	4.09 ± 5.16	-	P = 0.8

Severe CDI	16 (53.3%)	2 (50.0%)	0.9 (0.7-1.2)	P= 1
Patients outcome				
Deceased	1 (3.3%)	0 (0%)	-	P = 1
rCDI	4 (13.3%)	1 (25.0%)	1.1 (0.7-1.7)	P = 0.4
